# Chronic Sclerosing Osteomyelitis of Garré of the Femur in a 4-Year-Old Girl Caused by Coagulase-Negative Staphylococci: A Case Report

**DOI:** 10.3390/children13040451

**Published:** 2026-03-26

**Authors:** Nikolay Balgaranov, Stanimira Elkina, Irina Halvadzhiyan, Teodora Marinova-Bulgaranova

**Affiliations:** 1 Department of Pediatrics, Medical University—Pleven, 5800 Pleven, Bulgaria; irina.halvadjian@gmail.com; 2 Department of Microbiology and Virology, Medical University—Pleven, 5800 Pleven, Bulgaria; teodora.marinova@mu-pleven.bg

**Keywords:** chronic sclerosing osteomyelitis of Garré, femur, child, coagulase-negative staphylococci

## Abstract

**Background**: Chronic sclerosing osteomyelitis of Garré (CSO) is a rare, non-suppurative form of primary chronic osteomyelitis characterized by reactive periosteal bone formation and cortical thickening. It most commonly involves the mandibular bones, whereas long-bone localization is uncommon. **Material and Methods**: We report a 4-year-old girl who developed progressive right thigh pain and limping six months after receiving intramuscular ampicillin injections. Subsequent evaluation revealed femoral changes consistent with chronic sclerosing osteomyelitis. Surgical decompression and targeted antimicrobial therapy were performed. **Results**: Microbiological analysis of intraoperative specimens obtained prior to antibiotic therapy yielded *Staphylococcus epidermidis* (*S. epidermidis*) and *Staphylococcus capitis* (*S. capitis*). After three years of follow-up, the patient exhibited no functional impairment or growth disturbance of the affected limb. **Conclusions**: Although coagulase-negative staphylococci (CoNS) are commonly regarded as skin commensals, their repeated isolation from deep surgical specimens, together with clinical findings and response to treatment, raises the possibility of their involvement in the disease process in this case.

## 1. Introduction

Chronic sclerosing osteomyelitis of Garré (CSO) is an uncommon chronic, non-suppurative form of primary chronic osteomyelitis, characterized by reactive periosteal thickening and new bone formation in absence of overt purulence discharge [1]. This condition most frequently involves the mandible, particularly in children and young adults [2]. The predilection for the mandible has been attributed to its specific vascular supply, frequent odontogenic infections, and proximity to the oral microbiota. [3,4,5] In mandibular cases, chronic odontogenic inflammatory processes are commonly identified, with anaerobic bacteria most frequently implicated, whereas *Staphylococcus aureus* (*S. aureus*) and other staphylococci or streptococci are reported less often [6].

However, the etiology of CSO remains incompletely understood. Although a persistent infectious stimulus, most often odontogenic in origin, has traditionally been considered the principal contributing factor [2,4], additional etiological conditions have also been considered. These include periodontal disease [3], local trauma [7], altered host immune status [8], and complications after surgical management of fractures [9]. In rare instances, no identifiable precipitating factor is found, and the condition is considered idiopathic [10].

Extramandibular involvement, particularly in long bones, is rare and may be associated with minor trauma and presumed exogenous inoculation with low-virulence skin commensals, typically resulting in an indolent clinical course [9]. In this context, CoNS, such as *S. epidermidis* and *S. capitis*, are commonly regarded as components of normal skin flora; however, under certain conditions, they may be implicated in bone and joint infections. Their detection, therefore, requires careful interpretation to distinguish true pathogenic involvement from contamination [11,12].

We report an unusual case of femoral CSO in a child in whom *S. epidermidis* and *S. capitis* were isolated from intraoperative specimens, highlighting the diagnostic and etiological considerations associated with extra-mandibular disease.

## 2. Case Presentation

A 4-year-old girl was admitted to the University Pediatric Clinic, Medical University of Pleven, Bulgaria, due to persistent limping and pain in the right leg that had lasted for approximately six months. Past medical and family history were unremarkable.

One year before this hospitalization, she received a six-day ambulatory course of intramuscular ampicillin for a respiratory infection, administered alternately in both thighs. Approximately six months later, limping and pain in the right leg developed and persisted despite physiotherapy prescribed for presumed post-injection myalgia. Four months after onset, orthopedic examination and hip ultrasonography revealed no joint abnormalities. Two months later, due to increased pain and visible muscle atrophy, a radiograph of the right femur was obtained, revealing characteristic changes. The temporal association between the prior injections and subsequent symptom onset was documented in the medical history

On admission, the patient was in good general condition. Physical examination showed that the right thigh and calf circumferences were 2 cm smaller than the contralateral side and were associated with decreased muscle tone. Patellar and Achilles reflexes were hypoactive. Laboratory findings, including complete blood count, erythrocyte sedimentation rate (ESR), serum electrolytes, C-reactive protein (CRP), fibrinogen, liver transaminases, and antistreptolysin O (ASO) titer, were within the reference ranges. Tuberculin skin test and serological testing for syphilis were negative. Electromyography showed no evidence of peripheral nerve involvement.

**Figure 1:**
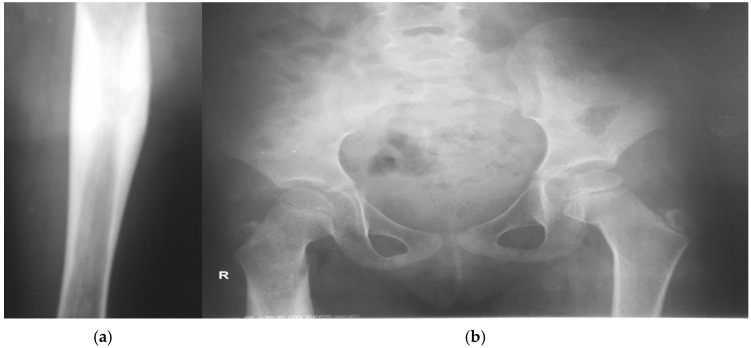
Radiography findings of the right femur: (**a)** cortical thickening and periosteal reaction along the diaphysis; (**b**) osteoporotic changes involving the femoral head, greater trochanter, and acetabulum.

The admission radiograph of the right femur demonstrated marked osteoporosis and a pronounced, multilamellar periosteal reaction at the junction of the upper and middle thirds of the femoral shaft, with involvement of the adjacent medullary canal and no evidence of osteolysis (Figure 1a). The left femur showed no pathological abnormalities. The radiographic findings of the right femur were suggestive of chronic osteomyelitis.

Additional images revealed osteoporotic changes involving the femoral head, greater trochanter, and acetabulum from the same side (Figure 1b).

The patient underwent surgical intervention consisting of opening and decompression of the sclerotic medullary canal. A cortical bone window measuring approximately 1 × 1 cm was created using trepanation. Tissue samples were obtained from the periosteum, cortical bone, and the medullary canal for histopathological and microbiological examination. Following sampling, a Redon suction drain was placed in the operative field.

**Figure 2:**
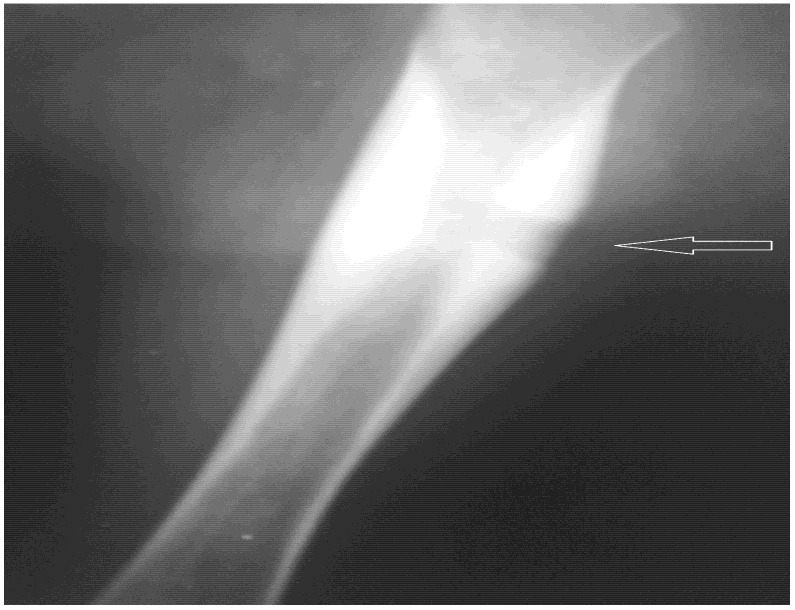
Postoperative radiograph demonstrating the cortical window and open medullary canal.

A postoperative radiograph demonstrated the cortical window created for biopsy and the opening of the medullary canal. (Figure 2).

**Figure 3:**
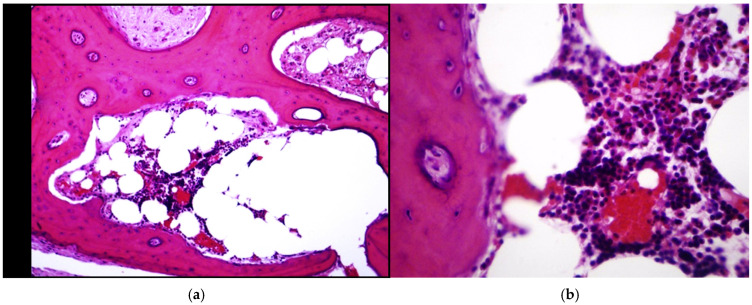
Histopathological findings: (**a**) Fibrous tissue with irregular bone trabeculae and sparse inflammatory infiltrate (hematoxylin and eosin, ×10); (**b**) Lymphoplasmacytic infiltrate with scattered neutrophils and no evidence of cellular atypia (hematoxylin and eosin, ×40).

Histopathological examination of the bone tissue obtained during open biopsy revealed fibrous connective tissue and fragments of compact bone with irregularly arranged and newly formed bone trabeculae. A sparse inflammatory infiltrate composed predominantly of lymphocytes and plasma cells with scattered neutrophils was observed between the trabeculae (Figure 3a). At higher magnification (×40), the inflammatory infiltrate was confirmed without evidence of cellular atypia (Figure 3b). According to the histopathological report, the findings were consistent with CSO.

Two samples for microbiological examination were obtained: one intraoperative biopsy specimen collected during open surgery and another sample obtained from the Redon suction drain on the first postoperative day. Cultures from both specimens yielded two coagulase-negative staphylococci—*S. epidemidis* and *S. capitis*. Species identification and antimicrobial susceptibility testing were performed using VITEK 2 system (bioMérieux, France). The results were interpreted according to CLSI guidelines valid at the time of testing. Both isolates were susceptible to all tested antibiotics, except *S. capitis*, which showed resistance to penicillin and intermediate susceptibility to Lincomycin. The susceptibility profile summarized in Table 1.

Based on these findings, postoperative antimicrobial therapy with intravenous vancomycin for two weeks was initiated, followed by ten days of oral cloxacillin according to the antimicrobial susceptibility results.

A control radiograph of the right femur obtained two months after the surgical intervention demonstrated the previously described multilamellar periosteal reaction along the diaphysis without evidence of osteolysis. The periosteal lamellae appeared nearly fused, forming a denser cortical structure. No new periosteal appositions were observed. The defect resulting from the biopsy showed indistinct margins and was partially filled with newly formed bone tissue. These findings were consistent with bone remodeling and regression of the inflammatory process.

During the three-year outpatient follow-up, the patient reported no pain or functional complaints.

Ewing sarcoma, osteoid osteoma, and specific infections such as tuberculosis and syphilis were considered in the differential diagnosis.

## 3. Discussion

CSO is most often associated with odontogenic infection of the mandible. However, the condition was originally described by Carl Garré in 1893 in association with proliferative periostitis of the tibia [13]. Current evidence suggests that this process may arise from chronic irritation or low-grade infection produced by microorganisms originating from the local flora of the skin or mucosal surfaces [7,14].

We present an unusual case of chronic osteomyelitis affecting the right femur. The patient’s medical history included a course of intramuscular injections administered approximately one year before presentation for a respiratory infection. Local trauma has been reported as a possible etiological factor in the development of CSO [7]. We therefore hypothesize that repeated intramuscular injections may have caused minor local trauma, which could have facilitated colonization of the affected bone by skin commensals and subsequently triggered a low-grade chronic infection. Smith et al. reported a case of acute osteomyelitis following an intramuscular injection of the human papillomavirus (HPV) vaccine [15]. Multiple local steroid injections have also been reported as a potential cause of calcaneal infection [16]. Furthermore, research by Yagdiran et al. [17] demonstrated the role of spinal injections in the establishment of infection, including CoNS.

The predominant microbial agents in mandibular cases are mixed anaerobes [18,19]. Our case yielded *S. epidermidis* and *S. capitis*, CoNS species that are typical skin commensals and therefore often regarded as culture contaminants. However, accumulating evidence indicates their role as opportunistic pathogens in bone and joint infections, particularly in chronic or low-grade infections [20,21,22,23]. According to Brooks et al., skin commensals such as CoNS, as well as *S. aureus*, are typical causative agents of infections following trauma and bone fractures [24]. S. epidermidis is the most common CoNS species isolated from clinical specimens [11]. Interestingly, data from the same study indicated that in France in 2005, *S. capitis* ranked third in frequency among isolates recovered from bone and joint samples. The chronic nature of the disease in the present case is supported by the prolonged clinical course, with symptoms evolving over approximately six months prior to diagnosis. Histopathological examination further demonstrated features consistent with a chronic inflammatory process, including the presence of fibrous tissue, lymphocytes, and plasma cells, with only a small number of polymorphonuclear leukocytes [20,21,25]. In addition, the radiographic findings—namely cortical thickening, multilamellar periosteal reaction, and involvement of the medullary canal—are consistent with imaging features described in chronic osteomyelitis [21,26].

The distinction between clinically significant pathogens and contaminating isolates remains a major challenge in clinical practice. To confirm the pathogenic role of CoNS repeated isolation of the same organisms is recommended [17,20,27]. In our case, *S. epidermidis* and *S. capitis* were independently isolated from two deep specimens: an intraoperative bone biopsy and a sample from a Redon suction drain collected on the first postoperative day. We acknowledge that microbial cultures obtained from postoperative drains may be prone to contamination. However, the concordant isolation of the same microorganisms from both specimens, together with the favorable clinical response to treatment, supports the hypothesis that these microorganisms may have played a potential etiological role. Furthermore, these observations are consistent with literature data indicating that CSO may be associated with low-virulence flora [20,21,22,23].

Several studies have shown that most CoNS infections are hospital-acquired or healthcare-associated [28,29]. Clonal dissemination of identical or closely related methicillin-resistant CoNS (MR-CoNS) strains within hospital environments has been demonstrated [30,31]. Recent reports indicate high rates of MR-CoNS in bloodstream infections, periprosthetic joint infections, and surgical site infections, reaching up to 80% [32,33,34,35]. In contrast, the *S. epidermidis* and *S. capitis* isolates in this case were susceptible to almost all tested antimicrobials. This finding may reflect their community origin and could be consistent with the introduction of the strains during intramuscular injections administered in the outpatient setting.

From a differential diagnostic perspective, Ewing sarcoma and osteoid osteoma were considered. However, histopathology demonstrated reactive bone formation with fibrous tissue and a sparse lymphoplasmacytic infiltrate without cytologic atypia, while imaging did not reveal a discrete nidus, making these neoplastic entities less likely [36,37]. Specific infections such as tuberculosis and syphilis were also considered. Although mycobacterial cultures were not performed, the absence of clinical features suggestive of tuberculosis, a negative tuberculin skin test, and the lack of granulomatous inflammation on histopathological examination argue against tuberculous osteomyelitis. In addition, the patient had no clinical stigmata or medical history suggestive of syphilis, and serological testing did not support this diagnosis [38,39,40].

Treatment of CSO is often challenging because the infection is indolent. Conservative therapy alone is frequently insufficient, whereas a combination of surgical intervention and targeted antibiotic treatment yields more favorable long-term outcomes [41,42]. In the presented case, surgical decompression of the medullary canal was performed in combination with targeted antibiotic therapy based on microbiological susceptibility testing. The favorable outcome was confirmed during follow-up by the absence of clinical symptoms together with radiographic evidence of bone remodeling.

## 4. Conclusions

This case highlights an unusual presentation of chronic sclerosing osteomyelitis of Garré involving the femur in a child. The combination of clinical findings, radiographic features, histopathological examination, and repeated isolation of coagulase-negative staphylococci from deep surgical specimens suggests a potential association between these microorganisms and the observed inflammatory process. The favorable clinical course following surgical decompression and targeted antimicrobial therapy was consistent with the hypothesis that the bone changes reflected a chronic infectious inflammatory process. Further studies are required to clarify the potential role of low-virulence skin commensals in the pathogenesis of extra-mandibular chronic sclerosing osteomyelitis.

## Figures and Tables

**Table 1:** Antimicrobial susceptibility of the isolates.

Antibiotic	*S. epidermidis*	*S. capitis*
Penicillin	S	R
Ampicillin	Not tested	Not tested
Cloxacillin	S	S
Carbenicillin	Not tested	Not tested
Piperacillin	Not tested	Not tested
Cephazolin/Cephalosporins	Not tested	Not tested
Gentamicin	S	S
Amikacin	S	S
Chloramphenicol	Not tested	Not tested
Erythromycin	S	S
Lincomycin	S	I
Clindamycin	S	S
Ciprofloxacin	S	S
Trimethoprim/Sulfamethoxazole	S	S
Rifampicin	S	S
Vancomycin	S	S

S—Susceptible, R—Resistant, I—Intermediate.

## Data Availability

All original data presented in this study are included in the article. Further inquiries can be directed to the corresponding author.

## Abbreviations

The following abbreviations are used in this manuscript:

CSO	Chronic sclerosing osteomyelitis of Garré
CoNS	coagulase-negative staphylococcal
MR-CoNS	methicillin-resistant CoNS
IDSA	According to the Infectious Diseases Society of America
ICM	International Consensus Meeting
